# Proteolytic cleavage and truncation of NDRG1 in human prostate cancer cells, but not normal prostate epithelial cells

**DOI:** 10.1042/BSR20130042

**Published:** 2013-06-11

**Authors:** Mohammad K. Ghalayini, Qihan Dong, Des R. Richardson, Stephen J. Assinder

**Affiliations:** *Discipline of Physiology, School of Medical Sciences, Bosch Institute, University of Sydney, Sydney, New South Wales, Australia; †Discipline of Medicine, Endocrinology Section and Bosch Institute, University of Sydney, Sydney, New South Wales, Australia; ‡School of Science and Health, University of Western Sydney, Penrith South, New South Wales, Australia; §Iron Metabolism and Chelation Program, Department of Pathology and Bosch Institute, University of Sydney, New South Wales, Australia

**Keywords:** CAP43, DRG1, NDR1, NDRG1, prostate cancer, TDD5, CBB, Coomassie Brilliant Blue, CIAP, calf intestinal alkaline phosphatase, DTT, dithiothreitol, E-cadherin, epithelial cadherin, FBS, foetal bovine serum, HRP, horseradish peroxidase, LDS, lithium dodecyl sulfate, NDRG1, *N*-myc downstream regulated gene-1, PNGase F, peptide *N*-glycosidase F, Phos-tag, phosphate-binding tag, PrEC, prostate epithelial cell, RT–PCR, reverse transcriptase–PCR, TGFβ, transforming growth factor β, λ-PPase, λ protein phosphatase

## Abstract

NDRG1 (*N*-myc downstream regulated gene-1) is a metastasis suppressor that is down-regulated in prostate cancer. NDRG1 phosphorylation is associated with inhibition of metastasis and Western blots indicate two bands at ~41 and ~46 kDa. Previous investigations by others suggest the higher band is due to NDRG1 phosphorylation. However, the current study using a dephosphorylation assay and the Phos-tag (phosphate-binding tag) SDS/PAGE assay, demonstrated that the 46 kDa NDRG1 protein band was not due to phosphorylation. Further experiments showed that the NDRG1 protein bands were not affected upon glycosidase treatment, despite marked effects of these enzymes on the glycosylated protein, fetuin. Analysis using RT–PCR (reverse transcriptase–PCR) demonstrated only a single amplicon, and thus, the two bands could not result from an alternatively spliced *NDRG1* transcript. Western-blot analysis of prostate cancer cell lysates identified the 41 kDa band to be a truncated form of NDRG1, with MS confirming the full and truncated proteins to be NDRG1. Significantly, this truncated protein was not present in normal human PrECs (prostate epithelial cells). Western-blot analysis using anti-NDRG1 raised to its N-terminal sequence failed to detect the truncated protein, suggesting that it lacked N-terminus amino acids (residues 1–49). Sequence analysis predicted a pseudotrypsin protease cleavage site between Cys^49^–Gly^50^. Such cleavage of NDRG1 in cancer cells may result in loss of NDRG1 tumour suppressive activity.

## INTRODUCTION

*NDRG1* (*N-myc downstream regulated gene-1*) is a member of the *NDRG* gene family [1]. All members of this group of proteins contain an α/β hydrolase fold, without a hydrolytic catalytic site [1]. The NDRG proteins are homologous to each other, sharing 52–62% sequence identity [2]. The *NDRG1* gene is mapped to human chromosome 8q24.3, an arm that is subject to prevalent genetic alterations [3]. Translation of the 3 kb *NDRG1* mRNA transcript leads to a 43 kDa protein composed of 394 amino acids that contains two key features which make it distinct from other NDRG family members, namely three tandem repeats (GTRSRSHTSE) near its C-terminal and a phosphopantetheine sequence [4,5].

*NDRG1* is regarded as a metastasis suppressor gene in cancers of the pancreas [6,7], colon [8], breast [9,10], cervix [11], ovaries [11] and prostate [10,12]. This is highlighted by the fact that overexpression of *NDRG1* in a prostate cancer cell line results in inhibition of metastasis *in vitro* and *in vivo* [12]. In prostate cancer tissue, NDRG1 levels are down-regulated and *NDRG1* expression has a significant inverse correlation with Gleason grade [12]. Furthermore, high NDRG1 levels correlate with an improvement in patient survival rate [12]. Hence, these clinical findings demonstrate that NDRG1 has profound metastasis inhibiting capabilities in prostate cancer and other tumours [7,8,10,12].

Recently, NDRG1 has been demonstrated to inhibit TGFβ (transforming growth factor β)-induced epithelial to mesenchymal transition by maintaining β-catenin and E-cadherin (epithelial cadherin) at the cell membrane [13]. NDRG1 has also been shown to affect the TGFβ pathway, leading to the up-regulation of two key tumour suppressor proteins, namely PTEN (phosphatase and tensin homologue deleted on chromosome 10) and SMAD4 [14]. In addition, NDRG1 was reported to inhibit the PI3K (phosphoinositide 3-kinase) and Ras oncogenic pathways [14]. However, further studies are required to fully elucidate the complex mechanism of action of NDRG1 in suppressing metastasis. Indeed, regulation of the protein's activity has yet to be understood.

NDRG1 appears to undergo phosphorylation at seven or more sites *in vitro* [15]. It has been suggested that the phosphorylation of NDRG1 inhibits the malignant progression of cancer by suppressing the activity of the NF-κB (nuclear factor-κB) signalling pathway, thereby reducing the expression of angiogenic CXC chemokines [16]. In addition, phosphorylated NDRG1 may play a role in controlling microtubule organization and cell abscission [17]. This suggestion is supported by the finding that phosphorylated NDRG1 co-localizes with γ-tubulin on centromeres and also at the cleavage furrow during cytokinesis [17]. However, additional investigation is needed to unequivocally determine whether the phosphorylation of NDRG1 confers its activity in terms of inhibiting metastasis.

Many studies examining different cell-types suggest that various primary antibodies used to detect NDRG1 recognize both non-phosphorylated and phosphorylated forms, resulting in two distinct bands detected by Western blotting [7,13,15–24]. Two reports have suggested that phosphatase treatment of cell lysates depletes phosphorylated NDRG1 detected by Western-blot analysis in HUVECs (human endothelial umbilical vein endothelial cells) [15] and MIAPaCa-2 pancreatic cancer cells [16]. While attempting to recapitulate these findings in prostate cancer cell lines, we demonstrated that a distinct truncated form of the NDRG1 protein exists. This post-translational modification event may decrease functional NDRG1 in prostate cancer cells that may affect its metastasis suppressor role.

## EXPERIMENTAL

### Cell culture

The human prostate cancer cell lines: DU145, PC3 and LNCaP, were from the A.T.C.C. The PC3MM/Tet-Flag-Drg-1 prostate cancer cell line was kindly provided by Dr K. Watabe (Southern Illinois University School of Medicine, IL, U.S.A.) [25]. Normal human PrECs (prostate epithelial cells) were from Lonza Clonetics.

The DU145, PC3, PC3MM and LNCaP cell lines were cultured in RPMI 1640 supplemented with l-glutamine (Invitrogen), 10% (v/v) FBS (foetal bovine serum; Invitrogen), 100 μg/ml of streptomycin and 100 units/ml of penicillin (Invitrogen). The human PrECs were cultured with the PrEGM™ Bullet Kit™ according to the manufacturer's instructions (Lonza Clonetics). Cells were cultured in a humidified atmosphere containing 5% CO_2_/37°C, as previously described [26].

### Western-blot analysis

Briefly, cells were lysed using lysis buffer (20 mM Tris/HCl, pH 7.5, 137 mM NaCl, 1% (v/v) Triton X-100, protease inhibitor cocktail [Complete™, EDTA-free; Roche Applied Science] and phosphatase inhibitor cocktail [PhosStop, Roche Applied Science]) and sonicated on ice. Bradford protein assay was used to determine protein concentration of lysates (Bio-Rad). Lysates were heated at 70°C with LDS (lithium dodecyl sulfate) sample buffer (Invitrogen) and DTT (dithiothreitol; Sigma–Aldrich) for 10 min. Protein samples (40 μg, unless otherwise stated) were then separated on NuPAGE 10% Bis-Tris SDS/PAGE (2.6% Bis-acrylamide) pre-cast gels (Invitrogen) and transferred for 7 min to PVDF membranes using the iBlot transfer system (Invitrogen). The membrane was blocked with 5% (w/v) non-fat dried skimmed milk powder in 0.1% Tris-buffered saline with 0.1% (v/v) Tween-20 for 1 h at room temperature. Membranes were then incubated overnight with either of the following antibodies (diluted in blocking solution): goat polyclonal anti-C-terminal NDRG1 (1:2500, Cat. No. ab37897; Abcam Inc.); rabbit polyclonal anti-N-terminal NDRG1 (1:2500, Cat. No. AP6935a; Abgent); or rabbit polyclonal anti-pNDRG1 (Ser330; 1:500, Cat. No. 3506; Cell Signaling Technology). Mouse monoclonal anti-β-actin primary antibody (1:10000 diluted in blocking solution, Cat. No. A5441; Sigma–Aldrich), used to indicate protein-loading, was incubated with membranes for 1 h at room temperature. The respective HRP (horseradish peroxidase)-conjugated anti-IgG secondary antibodies (1:2000 for N- and C- terminal NDRG1, 1:1000 for pNDRG1 and β-actin; all diluted in blocking solution) were then incubated for 1 h at room temperature. The blots were developed using ECL (enhanced chemiluminescence) (BM Mouse/Rabbit Chemiluminescence Western Blotting Kit, Roche) and visualized using a FluorChem® imager (Alpha Innotech Corp.). Protein size was determined by the AlphaEase molecular weight analysis tool (Alpha Innotech) implementing the known size of protein standards (Precision Plus All Blue™; BioRad) which were included in all analyses.

### Dephosphorylation assay

DU145 whole cell lysates (40 μg) were incubated in the presence or absence of either 500 units of λ-PPase (λ protein phosphatase; New England Biolabs) or 40 units of CIAP (calf intestinal alkaline phosphatase; New England Biolabs) for 2 h at 37°C. Negative controls included: (1) untreated lysates with or without PhosStop (Roche Applied Science); (2) lysates treated with a combination of exogenous phosphatase plus PhosStop; and (3) lysates treated with exogenous phosphatase, PhosStop and EDTA (87.5 mM) to completely inhibit phosphatase activity. Lysates were then incubated with sample buffer (Invitrogen) and DTT (Sigma–Aldrich) at 70°C for 10 min and analysed by Western blotting as detailed above. Positive controls to show active phosphatases included the loss of immunoreactive pNDRG1^Ser330^ and dephosphorylation of bovine milk α-casein (20 μg; diluted in lysis buffer), as shown by the Phos-tag (phosphate-binding tag) SDS/PAGE assay (see below).

### Phos-tag SDS/PAGE assay

The Phos-tag assay for visualization of phosphorylated proteins was performed according to the manufacturers’ instructions (Wako Chemicals Inc.) with some modifications. Briefly, 25 μM of acrylamide-pendant Phos-tag ligand (595 Da; Wako Chemicals) and 50 μM of MnCl_2_ were added to 10% polyacrylamide resolving gel solution before polymerization using routine procedures, while the stacking gel was made as previously described [26]. In all experiments, a 10% polyacrylamide resolving gel with omission of the Phos-tag molecule and MnCl_2_ was used as an internal control gel (standard SDS/PAGE). DU145 whole cell lysate (40 μg) in lysis buffer containing PhosStop was run using SDS/PAGE at 5 mA for approximately 16 h to ensure efficient separation and resolution of phosphorylated protein mobility-shifts [27]. Following separation, Mn^2+^-Phos-tag polyacrylamide gels were incubated for 1 h in transfer buffer with 1 mM EDTA and subsequently incubated in transfer buffer without EDTA for 10 min. Proteins were then electroblotted on to PVDF membranes (Roche) using standard procedures [26] and visualized for NDRG1 protein, as described above. The α-casein protein (20 μg; diluted in lysis buffer) and phosphatase-treated α-casein protein were used as a positive control to show an induced electrophoretic mobility-shift of phosphorylated α-casein relative to dephosphorylated α-casein.

### Deglycosylation assay

DU145 whole cell lysates (40 μg total protein) were incubated in the presence or absence of the N-linked glycosidase, PNGase F (peptide N-glycosidase F 1000 units; New England Biolabs). Separate lysates were also treated according to the manufacturers’ instructions with a protein deglycosylation cocktail containing *O*-linked glycosidases (New England Biolabs) and PNGase F. Treated lysates were incubated for 16 h at 37°C and subsequently analysed by Western blotting, as detailed above. Fetuin glycoprotein from FBS (20 μg diluted in lysis buffer) was used as a positive control to confirm glycosidase activity.

### Immunoprecipitation

To obtain sufficient levels of protein to allow for analysis by MS, NDRG1 was immunoprecipitated from cells overexpressing *NDRG1*. Namely, human prostate cancer cells (PC3MM) transfected with tetracycline-inducible (TET-ON) human NDRG1 (pcDNA5/TO/Flag-Drg-1) were used. PC3MM/Tet-Flag-Drg-1 selected cells were treated with 1.1 μg/ml tetracycline in RPMI 1640 for 24 h to induce *NDRG1* expression. Cells were then lysed using RIPA lysis buffer [65 mM Tris/HCl (pH 7.5), 150 mM NaCl, 5 mM EDTA, 1% (v/v) Triton X-100, 0.1% (w/v) SDS, 0.5% (w/v) sodium deoxycholate, 10% (v/v) glycerol, protease inhibitor cocktail [Complete™ EDTA-free; Roche Applied Science] and PhosStop and sonicated on ice. Cell lysates were incubated on a rotator for 3 h at room temperature with 1.5 mg of Dynabeads protein G (Invitrogen) that had been pre-incubated with anti-NDRG1 (1:12.5, C-terminal) antibody diluted in PBS with 0.02% (v/v) Tween-20 for 3 h at room temperature. The negative control went through the same procedure, but without antibody (No-Ab control). The Dynabeads were then washed three times with PBS followed by incubation at 70°C with LDS sample buffer (Invitrogen) and DTT (Sigma–Aldrich) for 10 min to elute the proteins bound to the Dynabead-protein G anti-NDRG1 antibody complex. Following this, eluted protein was separated by SDS/PAGE and the gel was subsequently fixed and stained with CBB (Coomassie Brilliant Blue) (R250) to detect protein and then used for MS analysis (as described below). Eluted protein was analysed by Western blotting to confirm immunoprecipitation of NDRG1.

### MS analysis

Samples were processed by the Sydney University Proteome Research Unit. Briefly, bands of interest were excised, destained and subjected to in-gel trypsin digestion at 37°C overnight. Peptides were spotted with an equal volume of α-cyano-4-hydroxycinnamic acid matrix and air-dried. MALDI-TOF (matrix-assisted laser-desorption ionization–time-of-flight) spectra were acquired using a Voyager DE STR mass spectrometer (AB Sciex). Peak lists were manually generated and used to search the Ludwig NR database (hosted by the Australian Proteomics Computational Facility, Melbourne, Australia) using Mascot (Matrix Science). The search criteria were set to: taxonomy unrestricted; enzyme specified as ‘trypsin’; one missed cleavage; tolerance±0.4 Da; and oxidation of methionine was a variable modification.

### RT–PCR (reverse transcriptase–PCR)

RT–PCR was performed as described previously [24] with minor modifications. Briefly, PCR was carried out using four sets of NDRG1 primers (Table 1) designed using the Primer3 website (http://frodo.wi.mit.edu/primer3/) to amplify four distinct regions of *NDRG1* by RT–PCR with the following cycle profile: one cycle of heat activation of the AmpliTaq Gold DNA polymerase (Promega) at 95°C for 1 min, then 39 cycles of 95°C for 30 s, 60°C for 30 s and 72°C for 30 s. The PCR was completed by one cycle at 72°C for 5 min. The PCR products were visualized on a 1% agarose gel by ethidium bromide. No-RT (no reverse transcriptase) and no-template were included as negative controls.

**Table 1:** Forward and reverse primer sets used to amplify *NDRG1*, expected amplicon sizes and exon coverage

Forward primer (5′→3′)	Reverse primer (5′→3′)	Amplicon size (bp)/ primer set/exon–exon
GTGAAGCCTTTGGTGGAGAA	AGAGAAGTGACGCTGGAACC	974/F1,R1/2–16
CTCTGTTCACGTCACGCTG	CTCCACCATCTCAGGGTTGT	349/F2,R2/4–8
CTGCACCTGTTCATCAATGC	AGAGAAGTGACGCTGGAACC	341/F3,R1/10–16
TCACCCAGCACTTTGCCGTCT	AGAGAAGTGACGCTGGAACC	757/F4,R1/5–16

## RESULTS

### The high molecular mass band of NDRG1 is not due to phosphorylation

**Figure 1:**
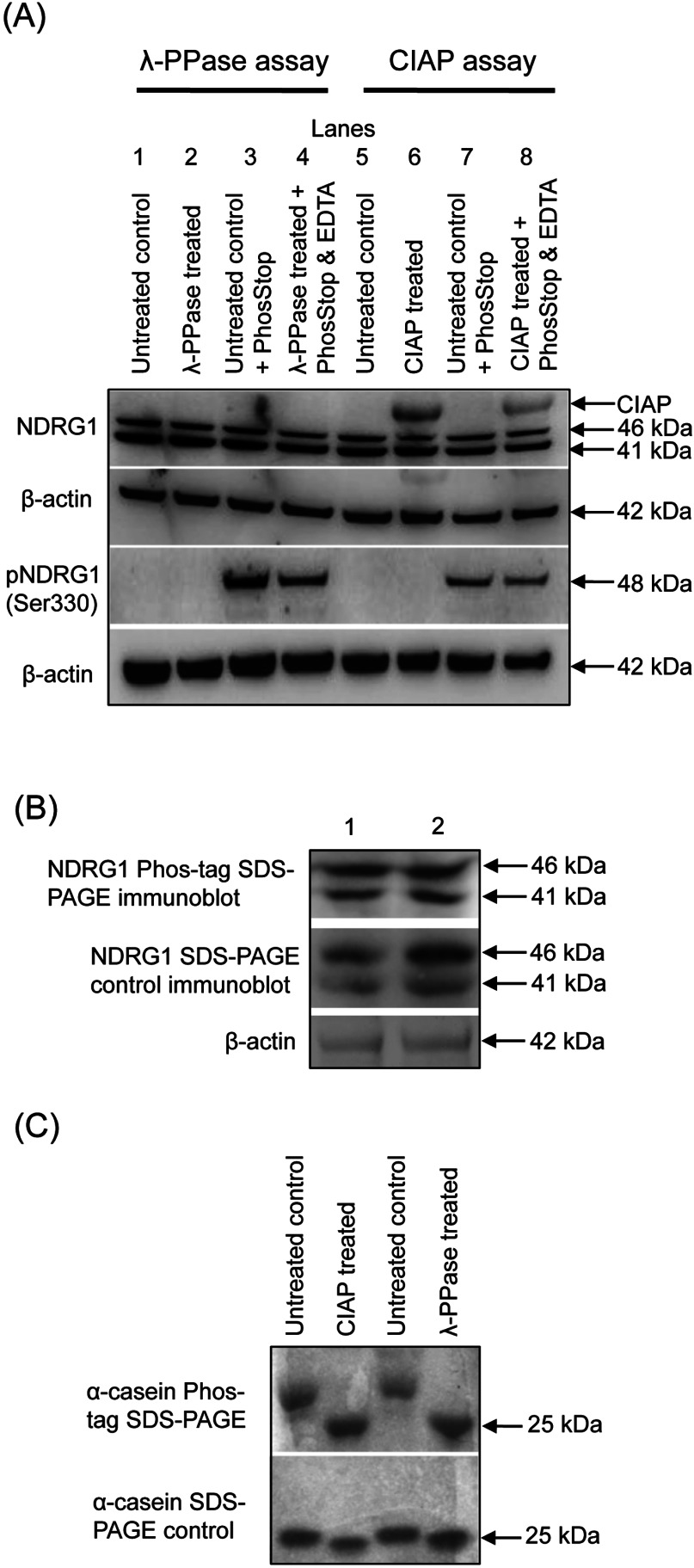
High molecular mass 46 kDa NDRG1 isoform identified in DU145 cells is not due to phosphorylation Analysis of (**A**) NDRG1 dephosphorylation assay using phosphatases and (**B, C**) Mn^2+^-Phos-tag SDS/PAGE assays. (**A**) Western blotting of DU145 whole cell lysates treated with or without λ-PPase or CIAP. Loss of immunoreactive pNDRG1^Ser330^ protein was used as a positive control to show active phosphatases. PhosStop and/or EDTA treatment was used to inhibit both endogenous (control lysates) and exogenous (λ-PPase or CIAP treated lysates) phosphatases. Blots were probed for β-actin which was used as a loading control. (**B**) A comparison of the NDRG1 Phos-tag SDS/PAGE immunoblot relative to the control NDRG1 SDS/PAGE immunoblot using DU145 cell lysates (duplicate samples are shown). β-actin was used as a loading control. (**C**) CBB staining of α-casein and dephosphorylated α-casein after performing Phos-tag SDS/PAGE or standard SDS/PAGE to confirm active phosphatases and Phos-tag efficacy. The figure is representative of three individual experiments.

We initially used DU145 cells for this investigation as two clearly defined NDRG1 bands were readily detected by Western blotting [13,20]. Indeed, upon visualization of Western blots with goat polyclonal antibody against C-terminal NDRG1, two protein bands were detected in DU145 cell lysates at ~41 kDa and ~46 kDa respectively (Figure 1A). In order to determine whether the 46 kDa band represented a phosphorylated isoform of the 41 kDa NDRG1 protein, we employed two different approaches, namely a dephosphorylation assay and the Phos-tag SDS/PAGE assay (see the Materials and methods section).

### Dephosphorylation assay

In these studies, whole cell lysates treated with or without λ-PPase or CIAP were utilized to assess NDRG1 phosphorylation status (Figure 1A). Loss of immunoreactive pNDRG1^Ser330^ protein was used as a positive control to show active phosphatases. PhosStop and/or EDTA treatment was used as they are both well known to inhibit both endogenous (untreated control lysates) and exogenous (λ-PPase or CIAP-treated lysates) phosphatases [27].

Treatment of DU145 lysates with either phosphatase, namely λ-PPase (Figure 1A, lanes 2 and 4) or CIAP (Figure 1A, lanes 6 and 8), failed to diminish the 41 or 46 kDa NDRG1 bands relative to the untreated controls (Figure 1A, lanes 1 and 5). These observations suggested that neither of these bands represented a phosphorylated isoform of NDRG1. Surprisingly, in CIAP-treated samples, an immunoreactive protein (~58 kDa; Figure 1A, lanes 6 and 8) was detected. It was not present in λ-PPase-treated or -untreated control lysates, nor was it detected with blots incubated with primary antibody only (results not shown). In fact, it was only observed following incubation with CIAP and in the presence of secondary anti-goat HRP-conjugated antibody. Hence, these results suggest this immunoreactive protein is of non-lysate origin and due to a non-specific interaction of the CIAP source with the secondary antibody.

Significantly the pNDRG1^Ser330^ band was not detected in DU145 untreated control lysates (i.e., no phosphatase inhibitor and no phosphatase added; Figure 1A, lanes 1 and 5) or λ-PPase- or CIAP-treated lysates without phosphatase inhibitor (Figure 1A, lanes 2 and 6, respectively). These observations suggested that the endogenous phosphatases in the control lysate and λ-PPase or CIAP, respectively, could remove phosphorylation of NDRG1 at Ser^330^. Hence, this activity is in marked contrast to the lack of effect of phosphatases on the 41 and 46 kDa NDRG1 bands described above. Consistent with this conclusion, in the presence of the phosphatase inhibitors, PhosStop and/or EDTA, both the untreated control lysates (Figure 1A, lanes 3 and 7) and the λ-PPase- or CIAP-treated lysates (Figure 1A, lanes 4 and 8) retained pNDRG1^Ser330^.

In summary, phosphatase treatment did not lead to depletion of the 41 or 46 kDa NDRG1 protein bands, while pNDRG1^Ser330^ immunoreactivity was abolished. Collectively, these observations suggested that the 41 and 46 kDa NDRG1 bands were not phosphorylated. To assess this finding further, a Phos-tag SDS/PAGE assay was performed.

### Phos-tag SDS/PAGE assay

As another method to assess NDRG1 phosphorylation, the Phos-tag SDS/PAGE assay was used. The Phos-tag ligand in the presence of Mn^2+^ binds to phosphate groups on proteins, increasing their mass and retarding their migration on SDS/PAGE, thereby inducing an electrophoretic mobility-shift [29]. In the presence of the Mn^2+^-Phos-tag (25 μM), there was no induced electrophoretic mobility-shift of the 46 kDa against the 41 kDa NDRG1 protein bands relative to control SDS/PAGE immunoblots conducted at the same time (Figure 1B). In the same experiments, as a relevant positive control, Phos-tag SDS/PAGE showed a pronounced electrophoretic mobility-shift of the phosphorylated α-casein protein (see Supplementary Figure S1A available at http://www.bioscirep.org/bsr/033/bsr033e042add.htm for phosphorylation sites) relative to α-casein protein dephosphorylated by either λ-PPase or CIAP (see upper panel, Figure 1C). In contrast, using standard SDS/PAGE, the mobility of purified α-casein protein showed a slight mobility shift to a lower molecular mass after incubation with λ-PPase or CIAP, consistent with dephosphorylation of α-casein (see lower panel, Figure 1C). Therefore, both the dephosphorylation and the α-casein Phos-tag SDS/PAGE assays confirm that the phosphatases are active and demonstrate the efficacy of the Phos-tag assay. Collectively, these data in Figures 1(A)–1(C) indicate that 46 kDa NDRG1 protein detected with an antibody against the C-terminal of NDRG1 in DU145 cells is not a phosphorylated NDRG1 isoform.

### The high molecular mass NDRG1 band at 46 kDa is not due to glycosylation

**Figure 2:**
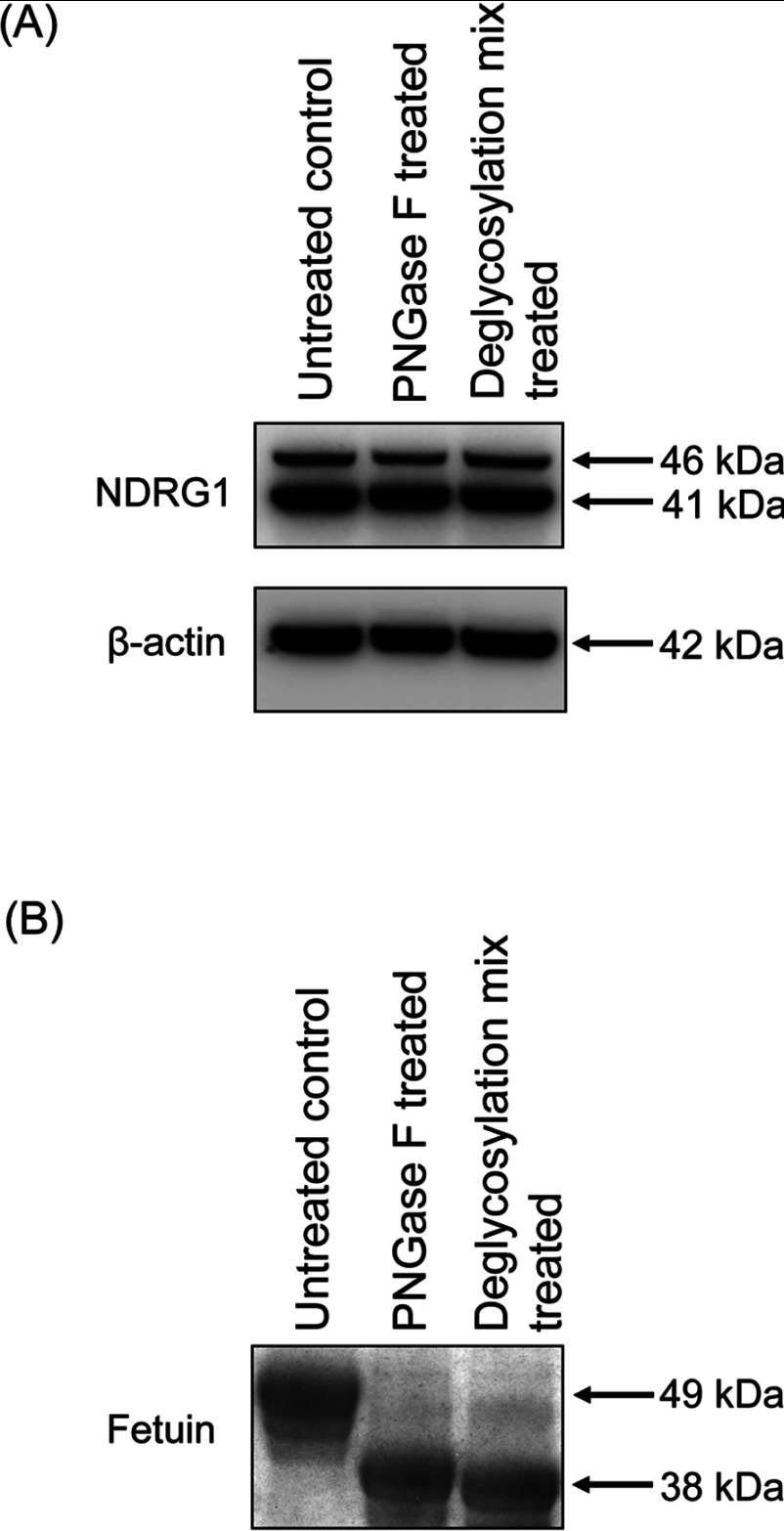
High molecular mass 46 kDa NDRG1 isoform of DU145 cells is not due to glycosylation (**A**) Western blotting of DU145 whole cell lysates treated with or without PNGase F and/or a deglycosylation mix composed of a PNGase F/O-linked glycosidase cocktail. β-actin was included as a loading control. (**B**) SDS/PAGE followed by CBB staining was used to visualize fetuin glycoprotein (positive control) treated with or without the glycosidases described in (**A**). The figure is representative of three individual experiments.

In order to determine whether the 46 kDa band was a glycosylated variant of the NDRG1 41 kDa band, we utilized a deglycosylation assay that implemented an N-linked glycosidase (PNGase F) and a PNGase F/O-linked glycosidase cocktail treatment of DU145 cell lysates. Neither treatment protocol resulted in mobility shift of the 46 kDa NDRG1 band relative to untreated control (Figure 2A). In the same set of experiments, we used the purified glycosylated protein, fetuin that acted as a positive control [30] (see Supplementary Figure S1C for glycosylation sites on fetuin). In contrast, to NDRG1, when fetuin was treated with PNGase F, it showed a clear electrophoretic mobility-shift relative to the untreated fetuin control (Figure 2B). In addition, when fetuin was treated with a protein deglycosylation mix containing a PNGase F/O-linked glycosidase cocktail, further slight electrophoretic mobility-shift relative to PNGase F-treated fetuin control was observed (Figure 2B). Therefore, these observations confirm active glycosidases in treated lysates. In summary, these results demonstrate that the high molecular mass NDRG1 protein is not a glycosylated NDRG1 isoform.

### Confirmation of NDRG1 protein *via* MS sequencing

**Figure 3:**
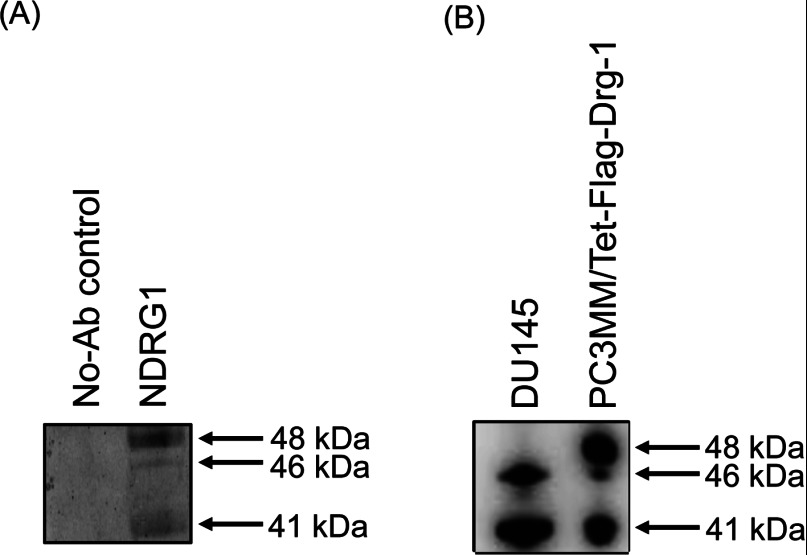
Identification of NDRG1 proteins by immunoprecipitation (**A**) Immunoprecipitation of the 41, 46 and 48 kDa proteins from PC3MM/Tet-Flag-Drg-1 cell lysates using the NDRG1 primary antibody implemented herein (i.e., see Figures 1A, 1B and 2A). Immunoprecipitates were separated using SDS/PAGE which were then CBB stained to visualize protein bands. (**B**) Western-blot analysis comparing the NDRG1 immunoprecipitated protein from DU145 and PC3MM/Tet-Flag-Drg-1 cell lysates. Results are representative of three individual experiments.

To verify that the NDRG1 primary antibody used in this study (i.e., the C-terminal antibody used above) was immunoreactive with the NDRG1 protein only, we set out to immunoprecipitate NDRG1 from lysates of the PC3MM/Tet-Flag-Drg-1 cell line overexpressing NDRG1. This cell line was specifically utilized to provide sufficient NDRG1 protein to sequence using MS. Notably, PC3MM cells also displayed two immunoreactive bands of 41 and 46 kDa (results not shown), while PC3MM/Tet-Flag-Drg-1 cells have an additional 48 kDa band corresponding to exogenous Flag-tagged NDRG1 (Figure 3). Three proteins immunoprecipitated with the NDRG1 primary antibody from PC3MM/Tet-Flag-Drg-1 cell lysates were identified to be NDRG1 proteins (Figure 3A and Table 2), with high MASCOT scores and sequence coverage ranging from 30 to 36%. The 48 kDa NDRG1 protein band represented the overexpressed exogenous Flag-tagged NDRG1, whereas the 41 and 46 kDa protein bands represented endogenous NDRG1 (Figure 3A). This conclusion was suggested by comparison with molecular masses of the immunoprecipitated NDRG1 protein from DU145 cells by Western-blot analysis (Figure 3B). These studies indicated that the full-length un-modified NDRG1 protein was represented by the 46 kDa high molecular mass NDRG1 band and that the 41 kDa NDRG1 band was a potential truncated protein.

**Table 2:** Identification of the 41, 46 and 48 kDa proteins as NDRG1 by MS analysis of immunoprecipated protein from PC3MM cells transfected with the NDRG1 expression vector, pcDNA5/TO/Flag-Drg-1

Mass (Da)			Residue number		
Peptide *M*_w_	Submitted	Matched	Start	End	Peptide sequence
48 kDa	2085.2	2085.1	2	19	SREMQDVDLAEVKPLVEK
	2101.2	2101.1	2	19	SREMQDVDLAEVKPLVEK+1 Met-ox
	1964.1	1964	54	70	GNRPVILTYHDIGMNHK
	1980.1	1980	54	70	GNRPVILTYHDIGMNHK+1 Met-ox
	1579.9	1579.8	95	110	SIIGMGTGAGAYILTR
	1595.9	1595.8	95	110	SIIGMGTGAGAYILTR+1 Met-ox
	1719.9	1719.8	161	174	EEMQSNVEVVHTYR
	1735.9	1735.8	161	174	EEMQSNVEVVHTYR+1 Met-ox
	2566.4	2566.2	175	196	QHIVNDMNPGNLHLFINAYNSR
	2585.3	2585.2	175	196	QHIVNDMNPGNLHLFINAYNSR+1 Met-ox
	1824.9	1824.8	269	284	YFVQGMGYMPSASMTR
	1840.9	1840.8	269	284	YFVQGMGYMPSASMTR+1 Met-ox
	1856.9	1856.8	269	284	YFVQGMGYMPSASMTR+2 Met-ox
	2375.2	2375.1	326	350	SHTSEGAHLDITPNSGAAGNSAGPK
46 kDa	1963.8	1964	54	70	GNRPVILTYHDIGMNHK
	1979.8	1980	54	70	GNRPVILTYHDIGMNHK+1 Met-ox
	1579.7	1579.8	95	110	SIIGMGTGAGAYILTR
	1595.7	1595.8	95	110	SIIGMGTGAGAYILTR+1 Met-ox
	1719.6	1719.8	161	174	EEMQSNVEVVHTYR
	1735.6	1735.8	161	174	EEMQSNVEVVHTYR+1 Met-ox
	2565.9	2566.2	175	196	QHIVNDMNPGNLHLFINAYNSR
	1824.6	1824.8	269	284	YFVQGMGYMPSASMTR
	1856.6	1856.8	269	284	YFVQGMGYMPSASMTR+2 Met-ox
	2374.9	2375.1	326	350	SHTSEGAHLDITPNSGAAGNSAGPK
41 kDa	1964	1964	54	70	GNRPVILTYHDIGMNHK
	1980	1980	54	70	GNRPVILTYHDIGMNHK+1 Met-ox
	1579.9	1579.9	95	110	SIIGMGTGAGAYILTR
	1595.9	1595.8	95	110	SIIGMGTGAGAYILTR+1 Met-ox
	2085	2085.1	142	160	ISGWTQALPDMVVSHLFGK
	2101	2101.1	142	160	ISGWTQALPDMVVSHLFGK+1 Met-ox
	1719.8	1719.8	161	174	EEMQSNVEVVHTYR
	2566.2	2566.2	175	196	QHIVNDMNPGNLHLFINAYNSR
	2582.2	2582.2	175	196	QHIVNDMNPGNLHLFINAYNSR+1 Met-ox
	1824.8	1824.8	269	284	YFVQGMGYMPSASMTR

Met-ox: methionine oxidation.

### *NDRG1* does not have an associated splice variant

**Figure 4:**
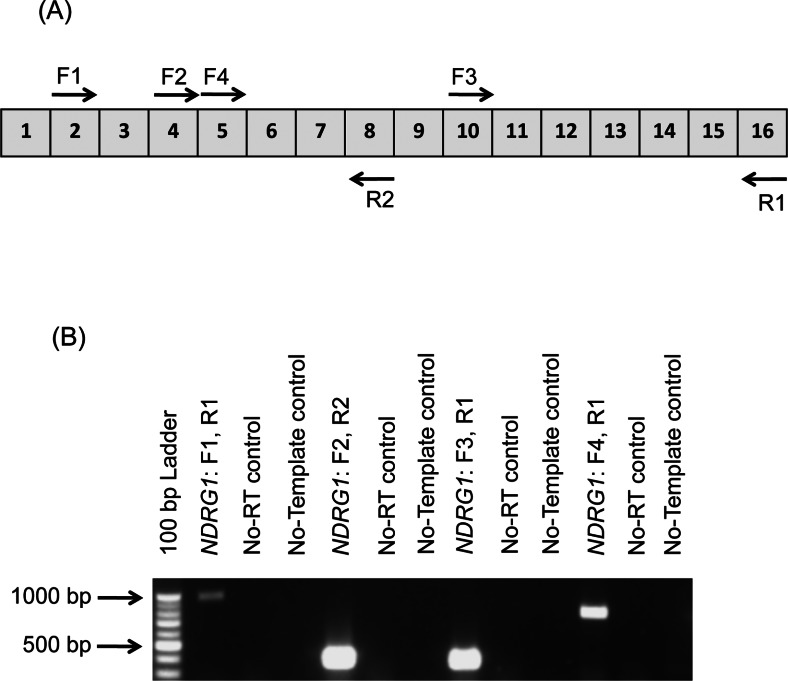
Distinct NDRG1 proteins are not due to alternative mRNA transcripts in DU145 cells (**A**) Four distinct *NDRG*1 primer (arrows) combinations specific to exon regions (grey boxes; Table 1) were designed for RT–PCR analysis assessing NDRG1 alternative splicing. (**B**) RT–PCR of DU145 cell mRNA was performed using the primer sets described in (**A**) in addition to No-RT and no-template controls. Amplicons generated correspond in size to those predicted. The figure is representative of two individual experiments.

MS analysis identified that the amino acid sequence coverage spanned the translated regions of exons 15 and 16 of *NDRG1* (Supplementary Figure S2 available at http://www.bioscirep.org/bsr/033/bsr033e042add.htm), thus confirming that the C-terminus of the 41 kDa NDRG1 isoform was intact. Therefore primer sets 1, 2, 3 and 4 (Table 1) were used to amplify exon regions 2–16, 4–8, 10–16 and 5–16 respectively of *NDRG1* in an attempt to identify *NDRG1* mRNA splice variants. RT–PCR analysis of DU145 total RNA showed that each of the four primer sets generated only a single amplicon corresponding to that of the predicted size (Figure 4). This indicated that the 41 kDa NDRG1 protein was not a result of translation of an alternatively spliced transcript of *NDRG1*.

### A truncated NDRG1 protein is present in human prostate cancer cell lines, but not in normal human PrECs

**Figure 5:**
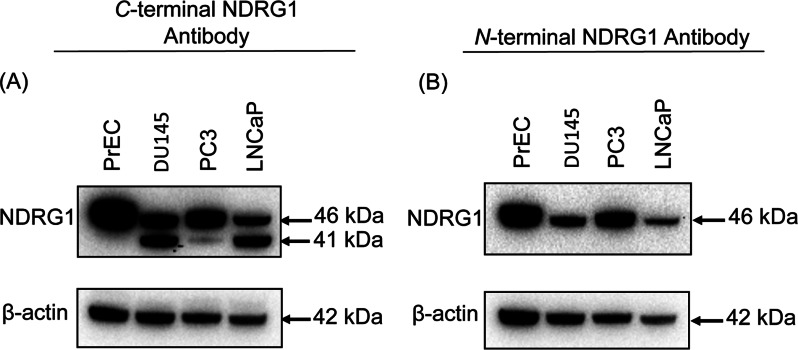
Western-blot analysis of normal human PrECs and metastatic prostate cancer cell lines, DU145, PC3 and LNCaP (**A**) A primary NDRG1 antibody to the C-terminus only detected an immunoreactive protein of 46 kDa in normal human PrEC, while both 41 kDa and 46 kDa immunoreactive species are present in lysates of DU145, PC3 and LNCaP prostate cancer cell lines. (**B**) A primary NDRG1 antibody directed to the N-terminus only detected an immunoreactive protein of 46 kDa in all cell-types analysed. β-actin was included as a loading control. The figure is representative of three individual experiments.

To determine whether the 41 kDa NDRG1 protein was a result of proteolytic cleavage of full-length NDRG1 at its N-terminus, Western-blot analysis was performed utilizing NDRG1 antibodies directed to either its C- or N-terminus. The presence of NDRG1 using these antibodies was compared in the human prostate cancer cell lines, DU145, PC3 and LNCaP, against primary cultures of normal human PrEC. The C-terminal NDRG1 antibody gave rise to two bands, one at 41 kDa and the other at 46 kDa in DU145, PC3 and LNCaP. However, only one NDRG1 band at 46 kDa was identified in the normal PrEC (Figure 5A). In contrast, the N-terminal NDRG1 antibody gave rise to only one 46 kDa band in primary cultures of normal PrEC and all prostate cancer cell lines (Figure 5B). This finding indicated that the 41 kDa NDRG1 protein band was not detected by the N-terminal antibody, suggesting that it lacked the N-terminal region and that a potential cleavage event had occurred.

### Prediction of a potential cleavage site in NDRG1 between amino acids Cys^49^–Gly^50^

**Figure 6:**
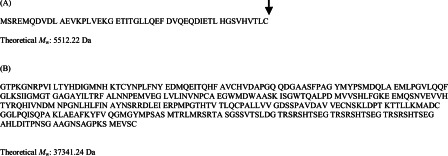
Hypothesized NDRG1 cleaved protein products (**A**) The 5.5 kDa product represents the N-terminal cleaved NDRG1 protein. The arrow indicates a cleavage point as predicted by PSORT II software (http://psort.hgc.jp). (**B**) The 37.3 kDa product represents the lower NDRG1 band on an immunoblot. Molecular mass of proteins was determined through the use of the Expasy MW tool (http://web.expasy.org/compute_pi/).

Analysis of the human NDRG1 protein sequence using PSORT II software (http://psort.hgc.jp) predicted a potential cleavage site between amino acids Cys^49^–Gly^50^ of NDRG1. This predicted cleavage of NDRG1 would lead to an approximate 5 kDa N-terminal by-product (Figure 6A), thereby decreasing the NDRG1 protein molecular mass from the predicted 43 kDa to 38 kDa (Figure 6B). Considering this, we consistently observed a 5 kDa difference between the two NDRG1 bands identified by the C-terminal anti-NDRG1 antibody, i.e. ~41 kDa and ~46 kDa (Figure 1A). This observation provides support to our hypothesis that NDRG1 is proteolytically cleaved between amino acids 49–50.

## DISCUSSION

Phosphorylation of NDRG1 has been associated with its metastasis inhibiting activities [16–17]. Indeed, NDRG1 undergoes phosphorylation at multiple sites [15,22,28], resulting in a phosphorylated isoform that is of a higher molecular mass. In contrast to past studies using other cell-types [15,16], in the current investigation, phosphatase treatment of DU145 prostate cancer cell lysates did not lead to depletion of the 46 kDa NDRG1 protein band (Figures 1A and 1B). However, pNDRG1^Ser330^ immunoreactivity was abolished by dephosphorylation with endogenous phosphatases in untreated control lysates and in lysates treated with exogenous phosphatases (λ-PPase and CIAP) in the absence of EDTA and PhosStop (Figure 1A). These studies suggested that the 46 kDa NDRG1 band was not due to phosphorylation of the 41 kDa band.

The experiments using phosphatases were then confirmed using the Phos-tag SDS/PAGE assay. The Phos-tag ligand in the presence of Mn^2+^ binds to phosphorylated serine, threonine or tyrosine residues and induces an electrophoretic mobility-shift when compared with standard SDS/PAGE [29]. In contrast with the phosphoprotein, α-casein, where a pronounced mobility-shift was identified by the Phos-tag SDS/PAGE assay (Figure 1C), no shift was observed for the NDRG1 bands (Figure 1B). Hence, contrary to previous reports using other cell-types [15–16], our results using the dephosphorylation and Phos-tag SDS/PAGE assays (Figures 1A–1C) indicate that the NDRG1 primary antibody was not reacting with a phosphorylated NDRG1 isoform. Thus, phosphorylation could not account for the 41 and 46 kDa bands observed in our studies.

The difference observed herein relative to previous investigations regarding NDRG1 phosphorylation may be attributable to different NDRG1 primary antibodies used. The NDRG1 primary antibody implemented in this investigation recognizes the GNSAGPKSMEVSC epitope, corresponding to the C-terminal amino acids 382–394 of human NDRG1. In the studies of Murakami et al. [16] and Masuda et al. [21], an antibody was used which recognized the TSEGTRSRSC epitope corresponding to the C-terminal tandem repeat region of NDRG1. It is possible that the antibody utilized by these latter investigators detects both the phosphorylated and non-phosphorylated NDRG1 proteins on Western-blot analysis of human MIAPaCa-2 pancreatic cancer cell line and renal cancer cell lines, 786-O and Caki-1. While this may explain the discrepancies between the current and previous studies, Piquemal et al. [5] used an NDRG1 primary antibody which recognized the (EGTRSRSHTS)_2_ epitope of C-terminal tandem repeat region for Western-blot analysis of U937 (human myelomonocytic cell line), Jurkat (human leukaemic T-cell line) and MCF-7 (human mammary carcinoma cell line) cell lysates. However, only one NDRG1 band of ~43 kDa was reported [5]. Collectively, these observations may be rationalized by suggesting that there is cell line specificity in terms of the presence of a higher molecular mass form of NDRG1.

Alternatively to phosphorylation, the ~46 kDa NDRG1 variant might be due to post-translational modification by glycosylation. However, the high molecular mass NDRG1 protein band at ~46 kDa was not depleted upon glycosidase treatment (Figure 2A), unlike that observed for the positive control, fetuin (Figure 2B). Significantly, the current study is consistent with the findings of Agarwala et al. [15] that indicated NDRG1 is a non-glycosylated protein.

It may be possible that one or more of the NDRG1 bands detected by Western blotting are due to non-specific binding of either primary or secondary antibodies. However, this interpretation is unlikely for several reasons. First, control experiments where primary antibody was omitted from Western-blot analysis gave no immunoreactive bands of 41 or 46 kDa. Second, pre-absorption of primary antibody with recombinant NDRG1 protein also resulted in the absence of any immunoreactivity on Western blottings (results not shown). Finally, MS analysis of immuno-purified cell extracts demonstrated both 41 and 46 kDa proteins to be NDRG1 (Table 2). Since the Flag-tag NDRG1 protein has a molecular mass greater than endogenous NDRG1 by 1–2 kDa, we surmised that the 48 kDa band detected in PC3MM/Tet-Flag-Drg-1 cells was the Flag-tag NDRG1 protein, while the 46 kDa band was the endogenous NDRG1 protein without any post-translational modifications. In contrast, the 41 kDa band represented a possible truncated protein. Furthermore, the juxtaposition of immunoreactive bands generated from DU145 and PC3MM/Tet-Flag-Drg-1 immunoprecipitated NDRG1 products on Western-blot analysis (Figure 3B), provides further support for the notion that the 48 kDa band in PC3MM/Tet-Flag-Drg-1 cells represented the Flag-tagged NDRG1 protein.

In the light of these findings, it was further hypothesized that the 46 kDa protein was the full-length un-modified NDRG1 protein and that the 41 kDa NDRG1 protein was either a result of the *NDRG1* transcript being alternatively spliced, or that NDRG1 protein was cleaved at its N-terminus. Considering this, all primer sets used in RT–PCR gave rise to a single product that corresponded in size to those predicted to be generated from the full-length, normally spliced transcript (Figures 4A and 4B). These results demonstrated that the 41 kDa NDRG1 band is unlikely to be a result of translation of a novel splice variant of the *NDRG1* gene transcript. These findings were consistent with previous studies showing that there are no spliced variants associated with *NDRG1*, as determined by Northern-blot analysis [9,21,31–36].

Western-blot analysis of PrECs and the three prostate cancer cell lines was then performed using two NDRG1 primary antibodies targeted to either the C-terminal epitope of NDRG1 (amino acids 384–392) or the N-terminal epitope of NDRG1 (amino acids 19–48). These studies demonstrated that the 41 kDa NDRG1 protein band was not detected by the N-terminal antibody, suggesting that it lacked the N-terminal region (Figure 5B). While the N-terminal NDRG1 antibody gave rise to the 46 kDa full-length NDRG1 protein in all cell lysates (Figure 5B), the C-terminal NDRG1 antibody detected the 46 kDa full-length NDRG1 protein and the 41 kDa cleaved NDRG1 protein in only the prostate cancer cell lines (Figure 5A). In contrast, only the 46 kDa full-length NDRG1 protein could be detected in PrECs when probed with the C-terminal NDRG1 antibody (Figure 5A). This observation suggests that the cleavage event was evident in prostate cancer cells, but not normal PrECs.

Of relevance, proteinases are central to the increased migration and invasive potential of prostate cancer cells, leading to prostate cancer metastases [37,38]. We speculate that the protease, pseudotrypsin, may be responsible for cleavage of the NDRG1 protein. This was predicted based on results using the peptide cutter tool in Expasy (http://web.expasy.org/peptide_cutter/), as pseudotrypsin is theoretically capable of cleaving at position Cys^49^–Gly^50^ of NDRG1. In fact, since tumour-associated trypsinogens are present in the DU145, PC3 and LNCaP prostate cancer cell lines [39], it is plausible that pseudotrypsin could cleave off NDRG1. Tumour-associated trypsin is expressed in a multitude of human cancers [40–44]. Therefore, the proteolytic cleavage of NDRG1 protein may not be just prostate-cancer specific. In support, studies using the same anti-NDRG1 antibody to the C-terminal identified two immunoreactive NDRG1 species in a range of human pancreatic cancer cell lines [13]. Therefore, proteolytically cleaved NDRG1 protein may be present in only certain types of cancer cells. Consistent with this hypothesis, in studies using C-terminal directed anti-NDRG1 antibodies, both single [5] and double [7,13,16,19–21] bands have been observed in a range of human tumour cell-types.

In summary, our data indicate that NDRG1 protein is being proteolytically cleaved at its N-terminus in the tumour metastasis-derived DU145, PC3 and LNCaP human prostate cancer cell lines. Initiation of metastasis is dependent on de-regulating the expression of both metastasis promoter and metastasis suppressor genes [37,45,46]. In human cancers, metastasis suppressor proteins such as E-cadherin and KiSS-1/metastin may undergo proteolytic cleavage, subsequently losing their functional capabilities [47–50]. This observation suggests that in addition to down-regulation of NDRG1 expression [12,25], an additional decrease of functional NDRG1 levels in prostate cancer cells may be mediated by proteolytic cleavage.
